# Transmission of a 2009 H1N1 Pandemic Influenza Virus Occurs before Fever Is Detected, in the Ferret Model

**DOI:** 10.1371/journal.pone.0043303

**Published:** 2012-08-29

**Authors:** Kim L. Roberts, Holly Shelton, Peter Stilwell, Wendy S. Barclay

**Affiliations:** Section of Virology, Department of Infectious Disease, Faculty of Medicine, Imperial College London, London, United Kingdom; University of Hong Kong, Hong Kong

## Abstract

During the early phase of the 2009 influenza pandemic, attempts were made to contain the spread of the virus. Success of reactive control measures may be compromised if the proportion of transmission that occurs before overt clinical symptoms develop is high. In this study we investigated the timing of transmission of an early prototypic strain of pandemic H1N1 2009 influenza virus in the ferret model. Ferrets are the only animal model in which this can be assessed because they display typical influenza-like clinical signs including fever and sneezing after infection. We assessed transmission from infected animals to sentinels that were placed either in direct contact or in adjacent cages, the latter reflecting the respiratory droplet (RD) transmission route. We found that pre-symptomatic influenza transmission occurred via both contact and respiratory droplet exposure before the earliest clinical sign, fever, developed. Three of 3 animals exposed in direct contact between day 1 and 2 after infection of the donor animals became infected, and 2/3 of the animals exposed at this time period by the RD route acquired the infection, with the third animal becoming seropositive indicating either a low level infection or significant exposure. Moreover, this efficient transmission did not temporally correlate with respiratory symptoms, such as coughs and sneezes, but rather with the peak viral titre in the nose. Indeed respiratory droplet transmission did not occur late in infection, even though this was when sneezing and coughing were most apparent. None of the 3 animals exposed at this time by the RD route became infected and these animals remained seronegative at the end of the experiment. These data have important implications for pandemic planning strategies and suggest that successful containment is highly unlikely for a human-adapted influenza virus that transmits efficiently within a population.

## Introduction

In 2009 the world experienced the first influenza pandemic of the 21^st^ century. During the early “containment phase” of the pandemic a variety of control measures were implemented, including identification and isolation of symptomatic individuals and their contacts, thermal screening at airports and other points of entry [1], and administration of prophylactic antiviral therapy to households with laboratory confirmed cases [2]. These control measures varied in their effectiveness but ultimately did not prevent the world wide dissemination of the virus.

When devising methods to reduce the spread of virus within a population it is critical to understand how onset of infectiousness correlates with onset of symptoms [3]. The proportion of transmission events that occur during the pre-symptomatic phase, between the time of exposure to the infectious agent and the onset of symptoms, is a key determinant of the success of reactive control measures. Containment of SARS in 2003 was successful in part because peak infectiousness followed peak symptoms [4]. Similarly, a recent publication showed that Foot and Mouth Disease Virus is predominantly spread from symptomatic infected cows and that investing in robust diagnostics to identify and remove these individuals could substantially mitigate the disastrous impact of an outbreak [5]. In contrast, it is presumed that some influenza virus transmission occurs from pre- or asymptomatic individuals [6] and this has been estimated for modelling purposes to be around one third of the transmission that occurs from symptomatic hosts [3]. Some anecdotal accounts of individual transmission events support the concept that pre-symptomatic individuals can infect susceptibles [7]. Similarly data from Lau et al. (2011) suggested that between 1 and 8% infections in the influenza season of 2008 may have occurred before onset of symptoms [8]. The early outbreaks of the 2009 H1N1 influenza pandemic provided an opportunity to address chains of transmission in the community. One study from Japan concluded that infectors transmitted disease to another person on the day of, or the day before onset of fever [9]. They described at least 5 instances of natural transmission occurring before onset of symptoms. In a separate account also from Japan, 3 clusters of pre-symptomatic transmission were characterized [10]. Moreover, studies to trace contacts of symptomatic individuals infected with pH1N1 virus who were exposed to large groups of people during train or bus travel or at a party suggest the rate of transmission during the symptomatic period was surprisingly low [11]–[13]. However, being sure about who infected who during transmissions in a natural setting is very difficult. To our knowledge, human volunteer studies that assess the timing of transmission in a controlled environment have not been reported. Indeed, a recent study demonstrated the difficulty of performing controlled human transmission experiments, showing that, even after serological screening to identify 12 naive susceptible volunteers, only 3 laboratory confirmed transmission events occurred [14].

Studies to assess the timing of influenza transmission in an experimental animal model are also sparse. Although the mouse is generally considered to be a less than optimal animal model for studies of this nature, Schulman and Kilbourne did report influenza transmission in mice. They exposed groups of naive mice to infected animals at different times following infection and showed a profound optimal timing for transmission at 24–48 hours post infection [15], [16]. Interestingly, the titres of infectious virus in the lungs of the donor mice did not correlate with transmissibility. Studies of naturally acquired human influenza infections have claimed various lengths of influenza contagiousness based on the presence of infectious virus or even of viral RNA detected in the nose of infected persons [17] but the mouse data suggests that presence of infectious virus alone does not necessarily predict transmissibility.

It is clear that further controlled studies that address whether pre-symptomatic individuals transmit influenza, and for how long they remain contagious, are required.

To address these questions we used the ferret model for influenza virus infection and transmission. Ferrets are an ideal model for this study because they are naturally susceptible to human-adapted influenza viruses and when infected they present the same range of clinical signs as humans, such as fever, lethargy, nasal discharge, sneezing and coughing.

During the past decade a body of work has used the ferret as a model host to study influenza virus transmission [18]–[20]. In all but one [21] of these studies reported so far, naive sentinel animals were exposed continuously to experimentally infected donor animals. This allowed for the identification of viruses that can or cannot be transmitted but it did not investigate for how long during infection the donor animals were contagious. In our own studies [22], we had noticed that the directly infected donor animals often shed virus in a biphasic manner. A peak of virus shedding occurred 1–3 days after inoculation, followed by a dip in virus load before a second burst of virus was excreted at around 5–7 days, followed by virus clearance. Although not universal, this bimodal shedding pattern has also been observed after infection of natural hosts of influenza such as horses, pigs or human volunteers [23]–[25] and has recently been modelled [26]. Here we examined whether the virus shed during these two different periods differed in its capacity to be transmitted to new hosts and whether the clinical signs displayed by the infected donor animals correlated with transmission.

## Results

### Contact transmission of a prototypic 2009 H1N1 pandemic virus

**Figure 1:**
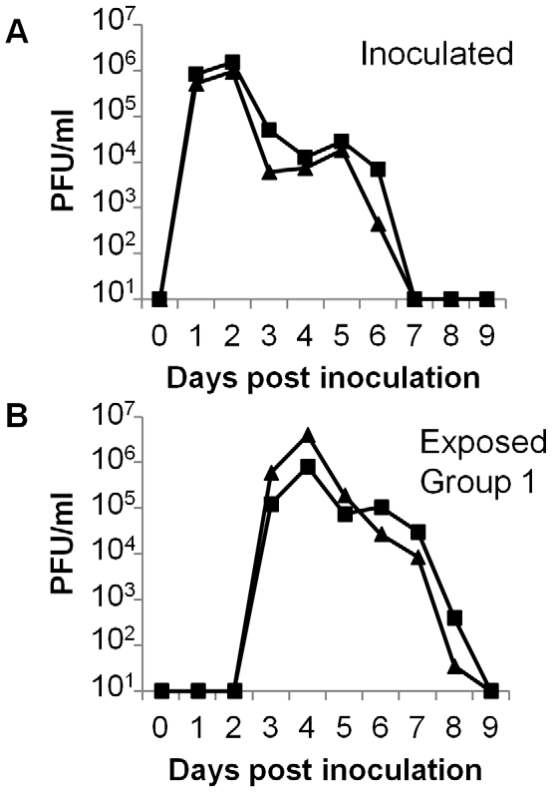
Transmission of pandemic H1N1 between inoculated and co-housed ferrets. Two ferrets were inoculated with 10^4^ PFU of E195 and 1 day pi a naive sentinel was co-housed with each inoculated donor. Daily nasal washes collected from inoculated (A) and exposed (B) animals were titrated by plaque assay. Ferrets in the same cage are indicated (triangles or squares).

To demonstrate the kinetics with which a prototypic influenza virus strain from the 2009 H1N1 pandemic, A/England/195/09 (E195), infected and transmitted between ferrets during continuous exposure, 2 animals were inoculated and 24 hours later were co-housed with naive sentinels. Virus was shed in the nasal wash of inoculated ferrets from days 1 to 6 post inoculation (pi) (Figure 1A), peaking on day 2 pi (1.5×10^6^ and 9.5×10^5^ PFU/ml). Both exposed sentinels (Group 1) shed virus in their nasal wash from days 2 to 7 post exposure (pe) (Figure 1B), with a similar viral titre on day 3 post exposure (8.0×10^5^ and 4.0×10^6^ PFU/ml).

### Contact transmission of influenza virus occurred both early and late during infection

**Figure 2:**
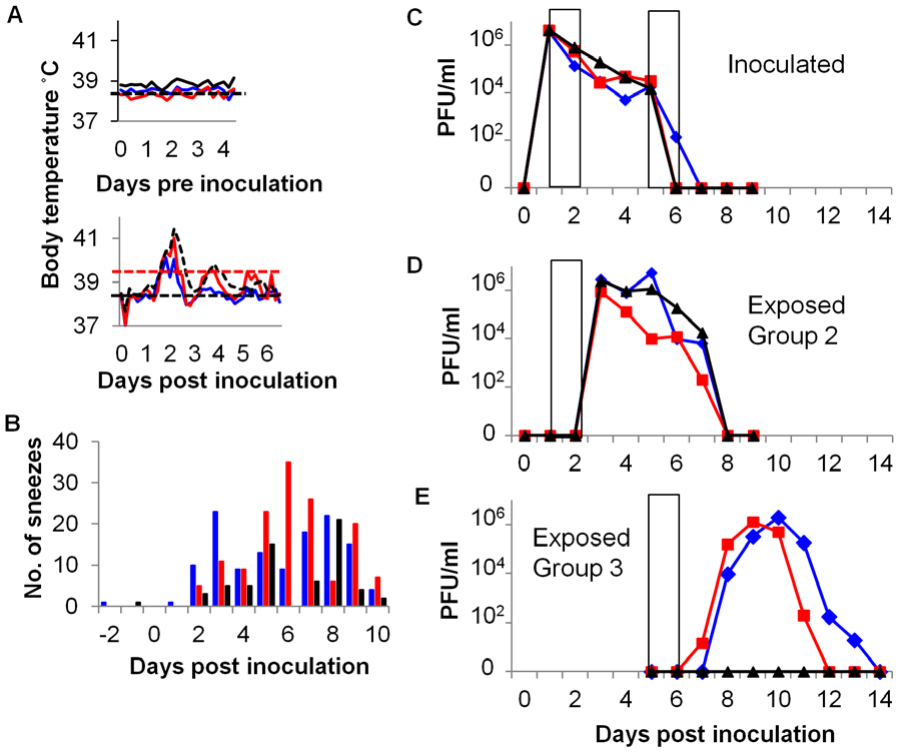
Transmission of influenza virus between co-housed ferrets, at both early and late periods during infection. Three ferrets were inoculated with 10^4^ PFU of E195. For 30 hours between days 1 and 2 pi each inoculated donor was co-housed with a naive sentinel (exposed group 2). A different group of naive animals were co-housed with the inoculated donors for 30 hours between days 5 and 6 pi (exposed group 3). (A) The core body temperatures of the inoculated donors were continuously monitored both before (above panel) and after (bottom panel) inoculation. The thin horizontal black line indicates baseline temperature (38.5°C) and the horizontal red line indicates fever (39.4°C). (B) The number of sneezes was recorded during a 15 minute observation period for the inoculated animals. Viral titres shed in nasal wash were determined by plaque assay: innoculated (C), exposed group 2 (D) and exposed group 3 (E). Ferrets in the same cage are indicated (red, blue and black). Exposure periods are indicated by the open bars.

To correlate the time of virus shedding with clinical signs, 3 animals were implanted with a continuous temperature telemetry transponder (Remo200, Remo Technologies Ltd, UK). After direct intranasal infection in the morning of day 0, the ferrets developed a fever towards the end of day 1 (beginning between 38 to 40 hours pi) that peaked around 48 hours pi and continued to the middle of day 2 (ending between 57 and 65 hours pi). A second, smaller fever peak occurred on day 3 (lasting between 85–97 hours pi) for 2 of the 3 inoculated donors (Figure 2A, bottom graph). Donor sneezing was first observed later than fever, on day 2 pi and was most pronounced from day 5 onwards. Respiratory clinical signs continued even after shedding of infectious virus had ceased (Figure 2B).

On the morning of day 1 pi, inoculated donors were each co-housed with a naive exposed sentinel for 30 hours (24–54 hours pi) (Figure 2C and D, Group 2). After the co-housing period the exposed ferrets were housed individually. The same inoculated donors were co-housed with a second group of sentinels for 30 hours beginning on the morning of day 5 pi (120–150 hours pi) (Figure 2C and E, Group 3), before all animals were individually housed again. All three inoculated animals shed virus in their nasal wash from day 1 pi until day 5 or day 6 pi (Figure 2C). Peak shedding (4×10^6^ PFU/ml, group mean) occurred on day 1 pi. Nasal washing was performed immediately prior to the two co-housing periods and it is of note that the donors were shedding approximately 2 logs less virus during the start of co-housing with Group 3 on day 5 (2×10^4^ PFU/ml, group mean), than with Group 2 on day 1 (4×10^6^ PFU/ml, group mean). All 3 exposed ferrets in Group 2 became infected and shed virus from 2 days pe until 6 days pe, with peak viral titres occurring on the day of onset (2×10^6^ PFU/ml, group mean). Only 2 of the 3 exposed animals within Group 3 became productively infected and the virologically negative animal did not seroconvert 3 weeks pe. Viral shedding from the two virus positive exposed animals in group 3 began 2–3 days pe but did not peak until day 4 pe (1×10^6^ PFU/ml) and 5 pe (2×10^6^ PFU/ml).

### Contact transmission occurred before the development of clinical signs

**Figure 3:**
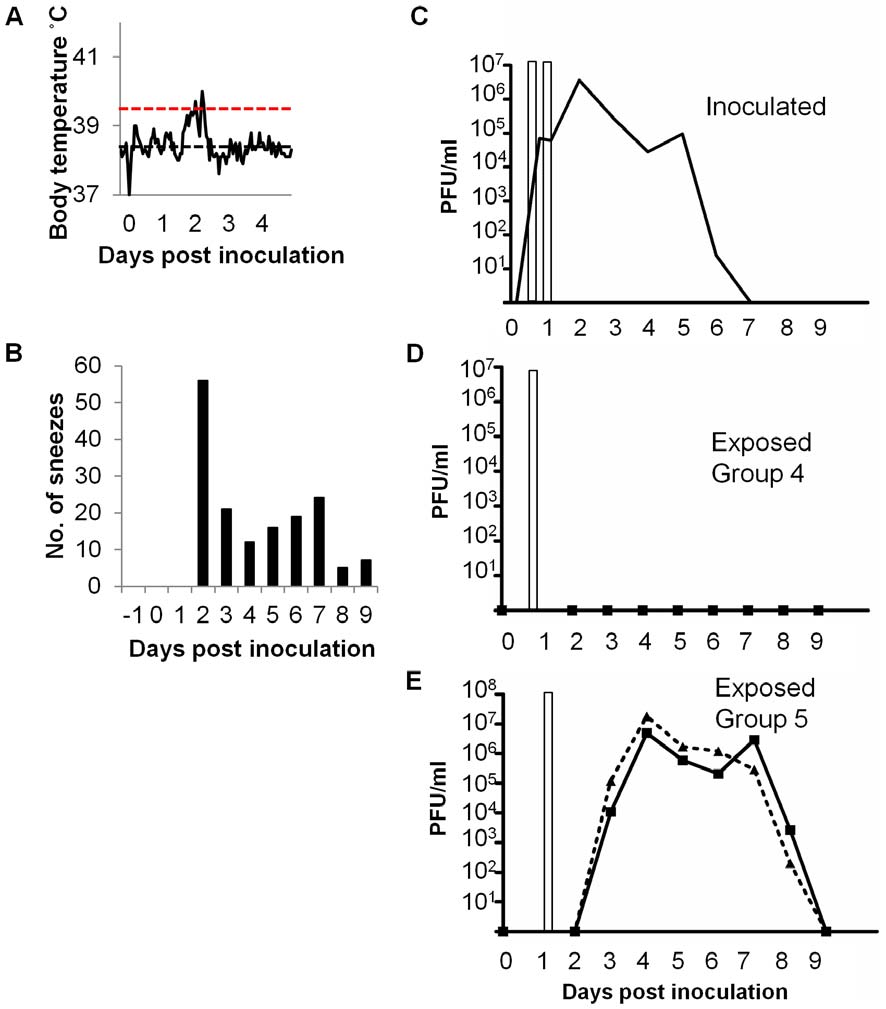
Transmission of influenza virus between co-housed ferrets exposed prior to clinical signs. One donor ferret was inoculated with 10^4^ PFU of E195. For 4 hours between 16 and 20 hours pi the inoculated donor was co-housed with 3 naive sentinels (exposed group 4), and for 4 hours between 24 and 28 hours pi with 2 other naive animals (exposed group 5). (A) The core body temperature of the inoculated donor was monitored by telemetry. The thin horizontal black line indicates baseline temperature (38.5°C) and the horizontal red line indicates fever (39.4°C). (B) The number of sneezes during a 15 minute observation period was recorded for the inoculated ferret. Viral titres shed from the nose were determined by plaque assay for the inoculated (C), exposed group 4 (D) and exposed group 5 (E). Exposure periods are indicated by the open bars.

To investigate whether transmission could occur before any measurable clinical signs, including fever which was the earliest clinical sign we detected, a donor animal implanted with a temperature telemetry transponder was co-housed with 3 sentinels (Group 4) for just 4 hours between 16 and 20 hours pi (7–11 am), then with another set of 2 sentinels (Group 5) between 24 and 28 hour pi (3–7 pm). Although ferrets are considered to be nocturnal animals, these ferrets were accustomed to interacting with handlers (for feeding, cleaning, playtime etc) during normal working hours and were therefore active during the day time. The donor was observed to be equally active and interactive during both co-housing periods. These co-housing periods were chosen based on the previous experiment (Figure 2) in which donors shed infectious virus in the nose at this time but did not display a fever until much later. Indeed in the inoculated donor in Figure 3, fever did not present until 45 hours pi (Figure 3A), and sneezing was not observed until 48 hours pi (Figure 3B), more than 24 hours after the end of the co-housing periods. No virus was detected in the nasal wash of the donor at 4 hours pi, but robust viral titres were recovered from a nasal wash taken at the end of the first exposure period (20 hours, 7×10^4^ PFU/ml) and at the end of the second exposure period (28 hour, 6×10^4^ PFU/ml) (Figure 3C). This suggests that Group 4 and 5 sentinels were exposed to secreted virus that was not residual inoculum (10^4^ PFU). None of the 3 Group 4 exposed sentinels became productively infected (Figures 3D) and sera at 21 days pe showed that they were serologically negative (microneutralisation <20) (Table 1). Both of the Group 5 exposed sentinels became infected and shed virus in nasal wash (Figure 3E).

**Table 1:** Summary of transmission events.

Exposed Group	Route of transmission	Length of exposure	Virus positive exposed sentinels	Serum positive exposed sentinels	Both virus and serum positive exposed sentinels
1	Contact	Continuous	2/2	NA	2/2
2	Contact	Early (30 h day 1–2 pi)	3/3	NA	3/3
3	Contact	Late (30 h day 5–6 pi)	2/3	0/1	2/3
4	Contact	Very early (4 h between 16–20 hpi)	0/3	0/3	0/3
5	Contact	Very early (4 h between 24–28 hpi)	2/2	NA	2/2
6	Respiratory droplet	Continuous	3/3	NA	3/3
7	Respiratory droplet	Early (30 h day 1–2 pi)	2/3	1/1	3/3
8	Respiratory droplet	Late (30 h day 5–6 pi)	0/3	0/3	0/3

NA, not applicable; h, hours; hpi, hour post inoculation of donor.

### Efficient respiratory droplet transmission of E195 with I219K HA mutation

**Figure 4:**
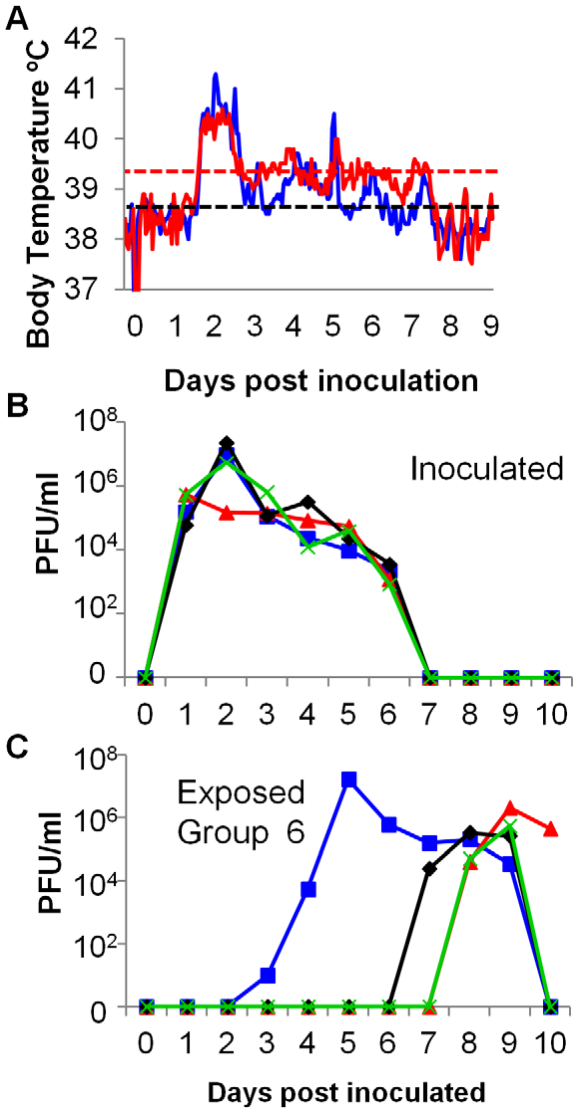
Respiratory droplet transmission of influenza virus. Four ferrets were inoculated with 10^4^ PFU of E195-I219K and 1 day pi naive sentinels (Exposed Group 6) were housed in cages adjacent to each donor. (A) The core body temperature of two of the inoculated donors was monitored by telemetry. The thin horizontal black line indicates baseline temperature (38.5°C) and the horizontal red line indicates fever (39.4°C). Virus shed in nasal wash from inoculated (B) and exposed (C) animals was titrated by plaque assay. Ferrets in adjacent cages are indicated (red, blue, black and green).

It is conceivable that transmission of virus through the air might require the generation of aerosols that contain infectious virus generated when a donor ferret sneezes. To test this idea we used a respiratory droplet transmission model. Initial experiments testing respiratory droplet transmission of wild type England/195/09 virus indicated that not all respiratory droplet sentinels became infected (data not shown) and this is in line with other reports in the literature that utilized the ferret model to study transmission of early isolates of the 2009 H1N1 pandemic virus, where transmission rates between 66% and 100% were reported [27]. In order to minimize animal numbers in further experiments, we engineered a recombinant virus in which the HA gene was mutated at the receptor binding site, changing an isoleucine at residue 219 to a lysine (E195-I219K), because this mutation was reported to increase sialic acid receptor binding affinity and transmissibility [28]. The virus shed from inoculated donors was similar in titre and kinetic profile to that shed from wild type E195 inoculated donors (compare Figures 1A, 2C, 3C and 4B) and infection with E195-I219K virus also induced a fever that started at 40 hours pi (Figure 4A). All 3 sentinel animals continuously exposed to inoculated donors through shared air alone contracted infection, although two of them did not begin to shed detectable virus from their nose until day 7 or 8 pe (Figure 4C).

### Respiratory droplet transmission occurred early but not late during infection

**Figure 5:**
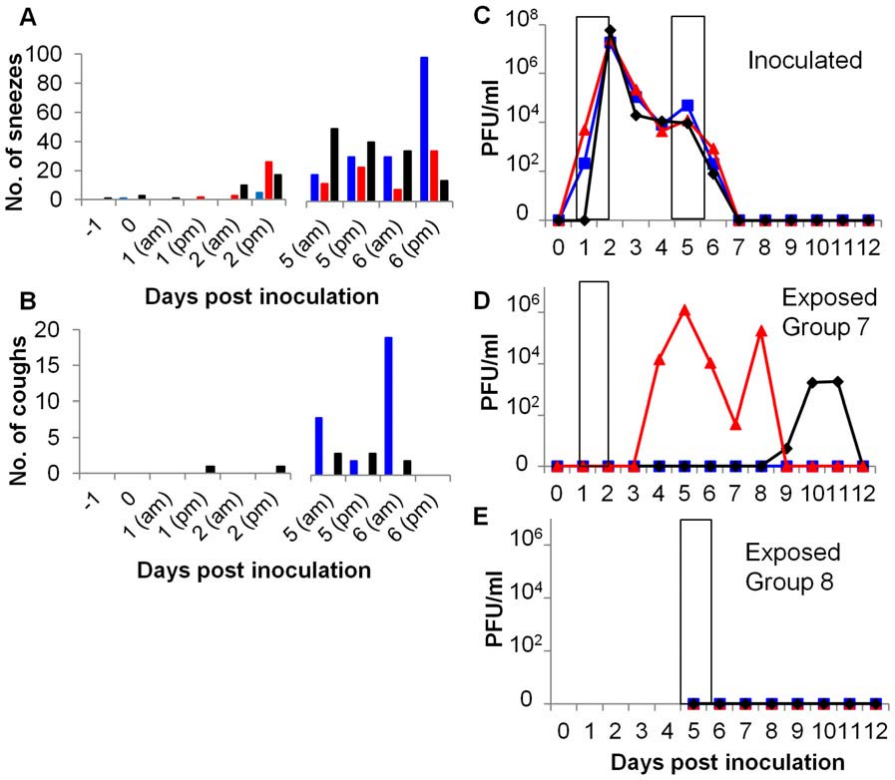
Respiratory droplet transmission of influenza virus, occurred before but not during clinical signs. Four ferrets were inoculated with 10^4^ PFU of E195-I219K. For 30 hours between days 1 and 2 pi a naive sentinel (exposed group 7) was housed in an adjacent cage to each inoculated donor. A different group of sentinels (exposed group 8) were exposed to air from the inoculated animals for 30 hours between days 5 and 6 pi. The number of sneezes (A) and coughs (B) in a one hour observation of the inoculated donors were recorded. Viral titres in daily nasal wash were determined by plaque assay: inoculated (C), exposed group 6 (D) and exposed group 7 (E). Ferrets in adjacent cages are indicated (red, blue and black). Exposure periods are indicated by the open bars.

Using this model of respiratory droplet transmission, we then tested transmission during the early phase of infection (air exposure for 30 hours between day 1 and 2 pi, 24–54 hours pi) or the period when respiratory signs were prominent (air exposure for 30 hours between days 5 and 6 pi, 120–150 hours pi). Figures 5A and 5B illustrate that respiratory signs were indeed rare during the first exposure period but common during the second after infection of donor animals with the recombinant E195-I219K virus. The virus shedding profile from the 3 inoculated donor animals was again biphasic (Figure 5C). Two of 3 sentinel animals exposed during the pre-symptomatic period acquired infection (Figure 5D) as detected by plaque assay. The third ferret was serologically positive 21 days pe, although it was virologically negative by plaque assay and quantitative PCR (data not shown), suggesting that transmission may have occurred but virus replication in the nose was at an undetectable level. In contrast none of the animals exposed to symptomatic ferrets between day 5 and 6 acquired the infection (Figure 5E). These exposed animals neither shed virus in their nasal wash (measured for up to 14 days pe), nor did they seroconvert 21 days pe.

## Discussion

The aims of this study were to investigate the temporal dynamics of influenza virus transmission within the ferret model; to determine whether influenza virus transmission was more efficient early or late during a controlled infection; and whether transmission correlated with observed respiratory clinical signs, such as coughs and sneezes. We found that contact transmission occurred following exposure during early or late phases of infection, but the shapes of the virus shedding profiles were different between the two groups of sentinels (Figure 2). Animals exposed during the late phase of donor infection showed an extended time to peak shedding of virus (Group 3) whereas animals who acquired their infections after exposure to donors on day 1–2 shed high titres of virus the following day after exposure. This might be due to a lower viral dose transmitted on day 5–6, even though the occurrence of sneezing by donors was higher during the second exposure interval than during the first.

Taken together these data imply that efficient transmission of influenza virus can occur before the onset of clinical signs. At 24–28 hours post inoculation, inoculated ferrets did not show respiratory signs nor was a temperature rise yet evident but during this short 4 hour window of exposure, all the contact sentinel animals acquired infection (Group 5, Figure 3). Recent data from Koster et al. (2012) also showed that respiratory droplet transmission could occur following a 3 hour exposure window 24 hours after infection of donors [21]. This particular exposure window may well represent the earliest time at which transmission occurs since none of the Group 4 animals exposed at the earlier time point of 16–20 hours post inoculation of donors were infected. Even though the inoculated animal was beginning to shed virus by the end of the very early exposure period, it is likely that the accumulated viral exposure during this period was insufficient to support transmission.

Whilst it is clear that different influenza strains might vary widely in the extent to which they induce clinical signs [29], in our own observations using at least 4 different human influenza viruses in ferrets we have not observed fever within the first 24 hours nor enhanced sneezing onset earlier than 2 or more days after infection ([22] and unpublished data). It has been mooted that sneezing is a prerequisite for transmission. We suggest instead that those viruses which replicate efficiently induce more damage to the respiratory tract and this leads to the sneezing response.

The issue of whether coughing and sneezing are required to expel infectious virus to the air has been nullified by several studies in which virus has been detected in exhaled air during tidal breathing [30], [31]. Expulsion of virus during normal breathing and also during sneezing from influenza infected ferrets was recently measured using an air analyzer sampler [32]. Even during normal breathing, infectious virus was shed into the air in droplets of less than 5 microns, a size that can penetrate the upper and distal airways. Moreover efficient influenza transmission occurs between guinea pigs even though they do not show clinical signs [33].

A second conclusion that can be drawn because of the limited exposure periods we employed in these experiments, is that the length of time between contracting virus and beginning to shed detectable infectious virus in the nose (the incubation period) may run beyond one week. Two respiratory droplet sentinels in Group 6 did not shed virus until almost a week after first exposure. Similar late shedding events have been reported for a variety of influenza viruses following respiratory droplet or even inefficient direct contact transmission (see for example [34]–[36]). Moreover the data in Figure 5 shows that when the exposure window was limited to day 1–2, one exposed sentinel in Group 7 did not shed virus until day 9, a full week after the known exposure period. Since Group 8 sentinels who were RD exposed at day 5–6 did not become infected, it is likely that those animals in Group 6 who acquired infection after continuous exposure were actually infected early during the exposure period but displayed a long incubation period. Therefore, in other studies where authors have suggested a correlation between sneezing and respiratory droplet transmission, they may, like us, have been observing viral shedding from sentinels following an extended incubation period. How such large and variable incubation periods impact on epidemiological models and affect the dynamics of onwards chain of transmission in a natural setting deserves further consideration.

Given the difficulty, both logistically and ethically, of performing human influenza transmission experiments, it is difficult to ascertain how well the ferret model replicates the dynamics of human influenza infection. Although the ferret model is the best that is currently available, studies like ours are limited by small animal numbers. It is also apparent that the severity and timing of illness induced in the ferret after inoculation differs depending on the route and dose of inoculums. Ferrets who have acquired their virus through the air following exposure to an infected animal may display a different pathogenic course than those who were directly inoculated [37]. Ideally the experiments we describe here would be better if performed using chains of transmission to better recapitulate the spread of virus through the community. However, reproducing chains of transmission in a laboratory setting will be logistically challenging. If symptom progression during influenza infection of ferrets is indeed comparable to the disease in humans [38], then our data suggest that control of influenza outbreaks may be even more difficult than public health planners have thus far perceived. Since transmission occurred with highest efficiency before overt clinical signs, even fever, were detected, control of influenza outbreaks by temperature screening at points of entry or the ‘stay at home’ policy may be unfeasible. On the other hand, the rather low transmission frequency late during infection, when coughs and sneezes induced by the damaged respiratory epithelium predominate, suggests that people might return to work soon after symptoms subside with little risk of onwards spread to colleagues. Indeed Donelly and co-workers showed that less than 5% household transmission events during the first pH1N1 2009 wave took place more than 3 days after onset of symptoms [39]. Furthermore, in a study of onwards transmissions from New York school children, more than half of household cases were detected within 3 days of onset of illness of the students suggesting that the serial interval was indeed short [40].

Our experimental data support these epidemiological observations, and reinforce mathematical models of pandemic spread that include a proportion of transmission occurring from pre-symptomatic individuals [3]. However it is likely that this proportion varies between influenza strains. It is noteworthy that a recent study of respiratory droplet transmission between ferrets also found that transmission of one prototypic pH1N1 2009 virus, Cal/04, occurred on day 1 and 3 after infection but not on day 5. However in the same study two other pH1N1 2009 virus strains did transmit from donors on day 5 after infection, although for these two viruses transmission was still more frequent on day 1 [21]. Thus it is likely that pre-symptomatic transmission frequency has been underestimated. The meta-analysis by Carrat et al. [41] of human volunteer influenza virus challenge studies concluded that peak viral shedding preceded peak symptoms by ∼1 day, corroborating the possibility that pre-symptomatic transmission can occur in humans as well as in the case shown here with the ferret model. We also suggest that the large amount of asymptomatic infection revealed by serological studies performed during the pandemic in 2009 supports the notion that transmission can occur in many settings in the absence of symptoms [42].

## Methods

### Viruses

Recombinant A/England/195/09 (H1N1) virus (E195) was generated using reverse genetics as previously described [43], [44]. E195-219K was constructed by substituting one amino acid in the HA gene (I219K) [28]. Virus stocks were produced via passage in Madin-Darby Canine Kidney (MDCK) cells. MDCK cells were maintained in DMEM (Gibco-Invitrogen, Inc.) supplemented with 10% fetal bovine serum (Biosera, Inc.), 1% penicillin/streptomycin (Sigma-Aldrich, Inc.) and 1% non-essential aas (Sigma-Aldrich, Inc.).

### Plaque Assay

Nasal wash samples were titrated on the day of collection. MDCK cells were inoculated with 100 µl serially diluted samples and overlaid with 0.6 % agarose (Oxoid) in supplemented DMEM (1× MEM, 0.21% BSA V, 1 mM L-Glutimate, 0.15% Sodium Bicarbonate, 10 mM Hepes, 1× Penicillin/Streptomycin, all Gibco and 0.01% Dextran DEAE, Sigma) with 2 µg trypsin (Worthington) ml−1 and incubated at 37°C for 3 days. The limit of virus detection in the plaque assays was 10 PFU/ml.

### Ferrets

All animal work was approved and licensed by the United Kingdom Home Office, PPL/70/6643. Female ferrets between 16–21 weeks old were obtained from a designated supplier. After acclimatisation, sera were obtained and all ferrets were negative for influenza antibodies by microneutralisation (MN) assay against E195 (pH1N1), A/Brisbane/10/07 (H3N2), and A/England/313/08, a seasonal H1N1 influenza virus.

### Continuous body temperature telemetry

Donor ferrets were surgically implanted (under anaesthetic, ketamine 22 mg/kg/xylazine 0.9 mg/kg followed by isoflurane) with a continuous temperature telemetry transponder (Remo200, RemoTech, UK), into the peritoneal cavity, 7 days before inoculation. Core body temperatures were recorded every minute and the hourly mean for each ferret calculated. The baseline core body temperature, 38.5°C±0.3°C standard deviation (SD), was calculated using the readings collected from 9 implanted, uninfected animals over 48 hours. Taking the baseline temperature plus 3 times the SD, fever was set as 39.4°C or above.

### Observation of respiratory symptoms

Donor animals were observed for 15 minutes (Figure 2 and 3) every day or one hour (Figure 5) during the morning and afternoon each day. All observations took place prior to nasal washing but approximately 15 minutes after the investigator entered the room to allow the ferrets time to settle. During the observations the number of sneezes and coughs were recorded. Ferrets sometimes had “sneeze fits” whereby multiple sneezes were recorded in very quick succession.

### Transmission experiments

Inoculated ferrets were lightly anaesthetised (ketamine 22 mg/kg/xylazine 0.9 mg/kg) and intranasally inoculated with 10^4^ PFU of virus in 200 ul PBS. For contact transmission, inoculated ferrets were co-housed with naive sentinel animals as previously described [22]. For respiratory droplet transmission, sentinel ferrets were housed in cages adjacent to the inoculated donors so that airflow delivered air from the inoculated donor cages across a short distance (25 mm) to the sentinel cages, through holes (5 mm diameter) in the cage. All animals were nasal washed whilst conscious, by instilling 2 ml PBS into the ferret's nose and collecting the expectorate. Nasal wash samples were titrated by plaque assay on MDCK cells. Strict procedures were followed to prevent aberrant cross-contamination between animals. Sentinel animals were handled before inoculated animals; all work surfaces and handlers' gloves were decontaminated between animals. Although in each experiment all animals were housed in the same room, after the controlled exposure period, sentinel animals were housed individually and on the opposite side of the room to the donors. Air flow within the room prevented cross-contamination of air between animals as proven by the placing of other naive sentinels in this area who remained virologically and serologically negative.

## References

[1] Cowling BJ, Lau LL, Wu P, Wong HW, Fang VJ, et al (2010) Entry screening to delay local transmission of 2009 pandemic influenza A (H1N1). BMC Infect Dis 10: 82. 20353566 10.1186/1471-2334-10-82 PMC3152767

[2] Ghani AC, Baguelin M, Griffin J, Flasche S, Pebody R, et al (2009) The Early Transmission Dynamics of H1N1pdm Influenza in the United Kingdom. PLoS Curr 1: RRN1130. 20029668 10.1371/currents.RRN1130 PMC2780827

[3] Fraser C, Riley S, Anderson RM, Ferguson NM (2004) Factors that make an infectious disease outbreak controllable. Proceedings of the National Academy of Sciences of the United States of America 101: 6146–6151. 15071187 10.1073/pnas.0307506101 PMC395937

[4] Anderson RM, Fraser C, Ghani AC, Donnelly CA, Riley S, et al (2004) Epidemiology, transmission dynamics and control of SARS: the 2002–2003 epidemic. Philos Trans R Soc Lond B Biol Sci 359: 1091–1105. 15306395 10.1098/rstb.2004.1490 PMC1693389

[5] Charleston B, Bankowski BM, Gubbins S, Chase-Topping ME, Schley D, et al (2011) Relationship between clinical signs and transmission of an infectious disease and the implications for control. Science 332: 726–729. 21551063 10.1126/science.1199884 PMC5844461

[6] Patrozou E, Mermel LA (2009) Does influenza transmission occur from asymptomatic infection or prior to symptom onset? Public Health Rep 124: 193–196. 19320359 10.1177/003335490912400205 PMC2646474

[7] Sheat K (1992) An investigation into an explosive outbreak of influenza. Communicable Disease New Zealand 92: 18–19.

[8] Lau LL, Cowling BJ, Fang VJ, Chan KH, Lau EH, et al (2010) Viral shedding and clinical illness in naturally acquired influenza virus infections. J Infect Dis 201: 1509–1516. 20377412 10.1086/652241 PMC3060408

[9] Yamagishi T, Matsui T, Nakamura N, Oyama T, Taniguchi K, et al (2010) Onset and duration of symptoms and timing of disease transmission of 2009 influenza A (H1N1) in an outbreak in Fukuoka, Japan, June 2009. Jpn J Infect Dis 63: 327–331. 20858998

[10] Gu Y, Komiya N, Kamiya H, Yasui Y, Taniguchi K, et al (2011) Pandemic (H1N1) 2009 Transmission during Presymptomatic Phase, Japan. Emerg Infect Dis 17: 1737–1739. 21888808 10.3201/eid1709.101411 PMC3322057

[11] Cui F, Luo H, Zhou L, Yin D, Zheng C, et al (2011) Transmission of Pandemic Influenza A (H1N1) Virus in a Train in China. J Epidemiol 21: 271–277. 21646746 10.2188/jea.JE20100119 PMC3899419

[12] Piso RJ, Albrecht Y, Handschin P, Bassetti S (2011) Low transmission rate of 2009 H1N1 Influenza during a long-distance bus trip. Infection 39: 149–153. 21340580 10.1007/s15010-011-0084-x PMC7099280

[13] Hermes J, Bernard H, Buchholz U, Spackova M, Low J, et al (2011) Lack of evidence for pre-symptomatic transmission of pandemic influenza virus A(H1N1) 2009 in an outbreak among teenagers; Germany, 2009. Influenza Other Respi Viruses 10.1111/j.1750-2659.2011.00251.x PMC5780667 21668675

[14] Killingley B, Enstone JE, Greatorex J, Gilbert AS, Lambkin-Williams R, et al (2012) Use of a human influenza challenge model to assess person-to-person transmission: proof-of-concept study. J Infect Dis 205: 35–43. 22131338 10.1093/infdis/jir701

[15] Schulman JL, Kilbourne ED (1963) Experimental Transmission of Influenza Virus Infection in Mice. Ii. Some Factors Affecting the Incidence of Transmitted Infection. J Exp Med 118: 267–275. 14074390 10.1084/jem.118.2.267 PMC2137714

[16] Schulman JL, Kilbourne ED (1963) Experimental Transmission of Influenza Virus Infection in Mice. I. The Period of Transmissibility. J Exp Med 118: 257–266. 14074389 10.1084/jem.118.2.257 PMC2137713

[17] De Serres G, Rouleau I, Hamelin ME, Quach C, Skowronski D, et al (2010) Contagious period for pandemic (H1N1) 2009. Emerg Infect Dis 16: 783–788. 20409367 10.3201/eid1605.091894 PMC2954014

[18] Herlocher ML, Elias S, Truscon R, Harrison S, Mindell D, et al (2001) Ferrets as a transmission model for influenza: sequence changes in HA1 of type A (H3N2) virus. J Infect Dis 184: 542–546. 11494159 10.1086/322801

[19] Maines TR, Chen LM, Matsuoka Y, Chen H, Rowe T, et al (2006) Lack of transmission of H5N1 avian-human reassortant influenza viruses in a ferret model. Proc Natl Acad Sci U S A 103: 12121–12126. 16880383 10.1073/pnas.0605134103 PMC1567706

[20] Lowen AC, Palese P (2007) Influenza virus transmission: basic science and implications for the use of antiviral drugs during a pandemic. Infect Disord Drug Targets 7: 318–328. 18220963 10.2174/187152607783018736

[21] Koster F, Gouveia K, Zhou Y, Lowery K, Russell R, et al (2012) Exhaled Aerosol Transmission of Pandemic and Seasonal H1N1 Influenza Viruses in the Ferret. PLoS One 7: e33118. 22509254 10.1371/journal.pone.0033118 PMC3317934

[22] Roberts KL, Shelton H, Scull MA, Pickles RJ, Barclay WS (2011) Lack of transmission of a human influenza virus with avian receptor specificity between ferrets is not due to decreased virus shedding, but rather a lower infectivity in vivo. J Gen Virol vir.0.031203–031200. 10.1099/vir.0.031203-0 21508186

[23] Baccam P, Beauchemin C, Macken CA, Hayden FG, Perelson AS (2006) Kinetics of influenza A virus infection in humans. J Virol 80: 7590–7599. 16840338 10.1128/JVI.01623-05 PMC1563736

[24] Saenz RA, Quinlivan M, Elton D, Macrae S, Blunden AS, et al (2010) Dynamics of influenza virus infection and pathology. J Virol 84: 3974–3983. 20130053 10.1128/JVI.02078-09 PMC2849502

[25] Brookes SM, Nunez A, Choudhury B, Matrosovich M, Essen SC, et al (2010) Replication, pathogenesis and transmission of pandemic (H1N1) 2009 virus in non-immune pigs. PLoS ONE 5: e9068. 20140096 10.1371/journal.pone.0009068 PMC2816721

[26] Pawelek KA, Huynh GT, Quinlivan M, Cullinane A, Rong L, et al (2012) Modeling within-host dynamics of influenza virus infection including immune responses. PLoS Comput Biol 8: e1002588. 22761567 10.1371/journal.pcbi.1002588 PMC3386161

[27] Maines TR, Jayaraman A, Belser JA, Wadford DA, Pappas C, et al (2009) Transmission and pathogenesis of swine-origin 2009 A(H1N1) influenza viruses in ferrets and mice. Science 325: 484–487. 19574347 10.1126/science.1177238 PMC2953552

[28] Jayaraman A, Pappas C, Raman R, Belser JA, Viswanathan K, et al (2011) A Single Base-Pair Change in 2009 H1N1 Hemagglutinin Increases Human Receptor Affinity and Leads to Efficient Airborne Viral Transmission in Ferrets. PLoS ONE 6: e17616. 21407805 10.1371/journal.pone.0017616 PMC3047569

[29] Coates DM, Sweet C, Quarles JM, Overton HA, Smith H (1985) Severity of fever in influenza: studies on the relation between viral surface antigens, pyrexia, level of nasal virus and inflammatory response in the ferret. J Gen Virol 66 (Pt 7) 1627–1631. 4020349 10.1099/0022-1317-66-7-1627

[30] Fabian P, McDevitt JJ, DeHaan WH, Fung RO, Cowling BJ, et al (2008) Influenza virus in human exhaled breath: an observational study. PLoS ONE 3: e2691. 18628983 10.1371/journal.pone.0002691 PMC2442192

[31] Stelzer-Braid S, Oliver BG, Blazey AJ, Argent E, Newsome TP, et al (2009) Exhalation of respiratory viruses by breathing, coughing, and talking. J Med Virol 81: 1674–1679. 19626609 10.1002/jmv.21556

[32] Gustin KM, Belser JA, Wadford DA, Pearce MB, Katz JM, et al (2011) Influenza virus aerosol exposure and analytical system for ferrets. Proc Natl Acad Sci U S A 108: 8432–8437. 21536880 10.1073/pnas.1100768108 PMC3100970

[33] Lowen AC, Mubareka S, Tumpey TM, Garcia-Sastre A, Palese P (2006) The guinea pig as a transmission model for human influenza viruses. Proc Natl Acad Sci U S A 103: 9988–9992. 16785447 10.1073/pnas.0604157103 PMC1502566

[34] Duan S, Boltz DA, Seiler P, Li J, Bragstad K, et al (2010) Oseltamivir-resistant pandemic H1N1/2009 influenza virus possesses lower transmissibility and fitness in ferrets. PLoS Pathog 6: e1001022. 20686654 10.1371/journal.ppat.1001022 PMC2912389

[35] Pappas C, Viswanathan K, Chandrasekaran A, Raman R, Katz JM, et al (2010) Receptor specificity and transmission of H2N2 subtype viruses isolated from the pandemic of 1957. PLoS One 5: e11158. 20574518 10.1371/journal.pone.0011158 PMC2888575

[36] Wan H, Sorrell EM, Song H, Hossain MJ, Ramirez-Nieto G, et al (2008) Replication and transmission of H9N2 influenza viruses in ferrets: evaluation of pandemic potential. PLoS One 3: e2923. 18698430 10.1371/journal.pone.0002923 PMC2500216

[37] Herfst S, Schrauwen EJ, Linster M, Chutinimitkul S, de Wit E, et al (2012) Airborne transmission of influenza A/H5N1 virus between ferrets. Science 336: 1534–1541. 22723413 10.1126/science.1213362 PMC4810786

[38] Reuman PD, Keely S, Schiff GM (1989) Assessment of signs of influenza illness in the ferret model. J Virol Methods 24: 27–34. 2760163 10.1016/0166-0934(89)90004-9

[39] Donnelly CA, Finelli L, Cauchemez S, Olsen SJ, Doshi S, et al (2011) Serial intervals and the temporal distribution of secondary infections within households of 2009 pandemic influenza A (H1N1): implications for influenza control recommendations. Clin Infect Dis 52 Suppl 1: S123–130. 21342883 10.1093/cid/ciq028 PMC3106264

[40] France AM, Jackson M, Schrag S, Lynch M, Zimmerman C, et al (2010) Household transmission of 2009 influenza A (H1N1) virus after a school-based outbreak in New York City, April-May 2009. J Infect Dis 201: 984–992. 20187740 10.1086/651145

[41] Carrat F, Vergu E, Ferguson NM, Lemaitre M, Cauchemez S, et al (2008) Time lines of infection and disease in human influenza: a review of volunteer challenge studies. Am J Epidemiol 167: 775–785. 18230677 10.1093/aje/kwm375

[42] Miller E, Hoschler K, Hardelid P, Stanford E, Andrews N, et al (2010) Incidence of 2009 pandemic influenza A H1N1 infection in England: a cross-sectional serological study. Lancet 375: 1100–1108. 20096450 10.1016/S0140-6736(09)62126-7

[43] Elleman CJ, Barclay WS (2004) The M1 matrix protein controls the filamentous phenotype of influenza A virus. Virology 321: 144–153. 15033573 10.1016/j.virol.2003.12.009

[44] Neumann G, Watanabe T, Ito H, Watanabe S, Goto H, et al (1999) Generation of influenza A viruses entirely from cloned cDNAs. Proceedings of the National Academy of Sciences of the United States of America 96: 9345–9350. 10430945 10.1073/pnas.96.16.9345 PMC17785

